# Copepods and ostracods associated with bromeliads in the Yucatán Peninsula, Mexico

**DOI:** 10.1371/journal.pone.0248863

**Published:** 2021-03-18

**Authors:** Nancy F. Mercado-Salas, Sahar Khodami, Pedro Martínez Arbizu

**Affiliations:** Senckenberg am Meer Wilhelmshaven, German Centre for Marine Biodiversity Research, Südstrand, Wilhelmshaven, Germany; University of Arkansas, UNITED STATES

## Abstract

A substantial fraction of the freshwater available in the Neotropical forests is enclosed within the rosettes of bromeliads that form small aquatic islands within a terrestrial landscape. These aquatic oases provide shelter, water, nutrients and resting of aggregation sites for several aquatic organisms, among them crustaceans. However, in comparison with the multitude of studies on open aquatic systems, our knowledge on crustaceans inhabiting semi-terrestrial habitats and phytotelmata is limited and their presence in such environments is poorly understood. The present study was carried out in two natural protected areas of the Yucatán Peninsula aiming to understand the diversity and dispersal strategies of crustaceans living in bromeliads. Sediment and water contained in four species of bromeliads have been collected in order to understand the diversity and dispersal strategies of crustaceans living in such habitats. From a total of 238 bromeliads surveyed, 55% were colonized by crustaceans. Sixteen copepod, three ostracod and one branchiopod species were recorded during this study, however only seven species are considered as true bromeliad inhabitants. Different degrees of association between crustaceans and bromeliad species were assessed with an indicator species analysis, where significant associations were found for all crustaceans. We found significant differences between bromeliad species and reserves and their associated fauna. In order to analyze the genetic diversity of this fauna, we sequenced several individuals of each species with two genetic markers (18S rRNA and COI mtDNA). Bayesian analyses and the Generalized Mixed Yule Coalescent method (GMYC), delimited 7 well supported species. A comparison of the dispersal strategies used by different species, including passive dispersal, phoretic behavior and active dispersal, is included. This study stresses the need of studying meiofauna of phytotelms, which could be used as an indicator of local diversity in Neotropical forests.

## Introduction

The Bromeliaceae is a family of vascular plants restricted to the New World, with the exception of a single species, *Pitcairnia feliciana*, distributed in West Africa [1, 2]. This family represents the largest Neotropical family of flowering plants, including 3140 valid species in 58 genera; its distribution ranges from Virginia, Texas, and California, USA, to northern Patagonia in Argentina. Bromeliads occupy a great variety of habitats, from granitic outcrops, coastal dune fields, mangroves, deserts and tropical rainforest to high altitude cloud forest [3–6]. These plants are known for their morphological and ecological plasticity shown by a large variety of life forms, ecological adaptations, pollination and dispersal modes, making them an important component of tropical ecosystems [7]. This family comprises mainly epiphytes, with most of them belonging to the phytotelmata type or tank bromeliads, forming rosettes (overlapping leaves) where the leaf litter and rain water accumulated are essential for the survival of many species, providing shelter, water, nutrients and resting or aggregation sites [7, 8]. These plants also contribute to the structural complexity of tree canopies, expanding the variety of microhabitats which are important for the establishment and maintenance of arthropod diversity in tropical ecosystems [7].

Research interest in phytotelmata micro-environments has recently increased because such environments are considered living laboratories which enable study of different natural processes such as colonization, dispersal, predator-prey interaction and competition [9]. Studies of phytotelmata fauna, including tree cavities, puddles in stumps of bamboo and similar grasses, bromeliad tanks, pitcher plants, water filled coconut husks, and *Heliconia* flowers are typically dominated by larval stages of insects, yet several groups of invertebrates can also be found [10, 11]. Among these invertebrates are freshwater crustaceans such as Ostracoda, Copepoda, and Anomopoda, which have been recognized as passive dispersers that move among habitat patches using other animals as vectors [10, 12, 13]. In the last global overview of phytotelmic crustaceans, 108 species of crustaceans (Copepoda, Ostracoda, Anomopoda and Decapoda) were identified in studies from 1886–2010, and other unpublished data [10]. Ostracods are the most common crustaceans found in phytotelmata, where currently 16 species have been described from this habitat, with 11 species being exclusive to bromeliads. The most common bromeliad ostracods belong to the genus *Elpidium* Müller, 1880, recorded in Florida, Mexico, Central America, Brazil and the Caribbean [13].

Copepoda are by far the most diverse group of crustaceans found in phytotelmata, with more than 60 species recorded from these habitats. About half of these records refer to species inhabiting tank bromeliads including 13 species of Harpacticoida and 22 species of Cyclopoida, however studies of copepod fauna have been more related to casual findings of other groups than studies of their bromeliad habits. Most of these studies have been restricted to new species descriptions from bromeliads, and there is little information about their ecology, dispersal, genetic variation or their connectivity among plants [14].

Mexico represents the diversification center for some bromeliad groups. Currently 422 species consisting of 19 genera have been recorded [15, 16] with *Hechtia*, *Pitcairnia*, *Tillandsia* and *Aechmea* as the most diverse groups in the country [17]. The study of phytotelmata-associated arthropods in Mexico has been insect-arachnid dominated, as it is in other countries [18]. However, despite the lack of studies about crustaceans, Mexico possesses the highest crustacean richness (8 spp.) worldwide recorded from an endemic bromeliad of the dry forest in Aguascalientes state [10]. Additionally, two more crustacean species were recorded from Mexican bromeliads during that study. Further records of species inhabiting phytotelmata in Mexico are the copepod *Olmeccyclops veracruzanus* (Suárez-Morales, Mendoza & Mercado-Salas, 2010) from the bromeliad *Tillandsia heterophylla* found in a cloud forest in Veracruz [19] and recently the cladoceran *Disparalona hamata* (Birge, 1879) inhabiting *Tillandsia aguascalentensis* from Aguascalientes state [20].

In the Yucatán Peninsula (YP) there are 31 species of Bromeliacea recorded from which about 87% are epiphytes and 13% are terrestrial, sub-terrestrial or lithophytes, distributed in different vegetation types [17, 21]. Knowledge of continental crustaceans of the YP includes records of 212 species of which 48 are considered endemic [22]. However, studies on crustaceans associated with bromeliads have not been performed in this region of Mexico. Due to the permeability of the substrate present in the YP, no surface streams or rivers exist with the exception of the Hondo River, the major water bodies in the YP are underground or temporary [23–25]. Thus it is probable that epigeous crustacean species have been forced to colonize new environments that provide suitable aqueous environments. The YP represents an excellent area to study bromeliad-associated crustaceans for species richness, patterns of colonization, and habitat preferences because this area is not influenced by river flooding or major water bodies. Thus, we hypothesize the presence of crustaceans in bromeliads can be only attributed to evolutionary process and not to a casual event as in other geographic areas.

## Materials and methods

**Fig 1:**
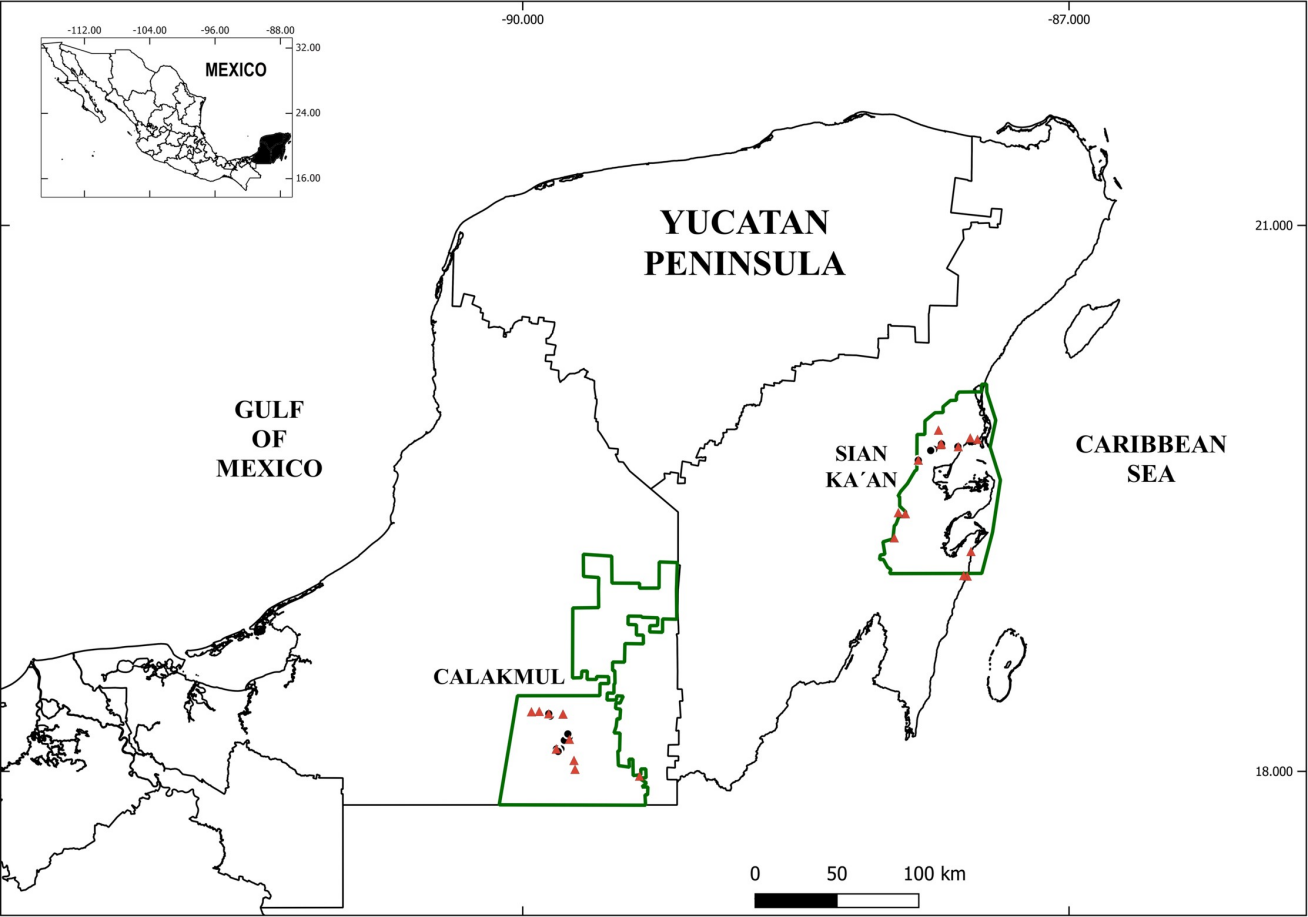
Sampling localities in Calakmul and Sian ka´an Biosphere Reserves. –Symbols: black circles- localities sampled during 2014; red triangles- localities sampled during 2015.

Two Biosphere Reserves, Sian Ka´an and Calakmul, were sampled for this study (Fig 1) (collection permit: PPF/DGOPA-003/15 SEMARNAT-CONAPESCA, Mexico). These reserves represent the most extensive areas of well-preserved tropical forest in Mexico. Biosphere Reserve Sian Ka´an comprises 528,000 ha of both terrestrial and marine habitats. The reserve occupies a partially emerged limestone plateau that gradually descends to the sea, forming a gradient from dry to seasonally flood areas. The Biosphere Reserve Calakmul comprises 723,185 ha of terrestrial habitat including a combination of high and medium forest with seasonal flooded lowlands and aquatic vegetation [26]. It hosts 1353 “aguadas” or temporary or permanent pools and a Paleocene aquifer with a depth to the phreatic zone between 60 and 165 m [27].

Samples were collected in September 2014 and September-October 2015. Seventeen localities were established in Calakmul Reserve and sixteen in Sian ka´an Reserve and two localities in Calakmul and three in Sian ka´an were sampled both years (details in S1 Appendix). For each locality a 90m transect was established and samples were taken at 0 (S1), 30 (S2), 60 (S3) and 90 (S4) meters from the initial point, and in some localities an epigeous waterbody was taken as the initial point of sampling. At each station we collected water accumulated between bromeliad leaves, as well as from bromeliads in the surrounding 2m of the collection point. Organisms from bromeliads were collected directly by pipetting the water accumulated in the central pool and washing the outer leaves of the plant in order to collect all water and sediments contained in the bromeliads (modified from [28]). For the bromeliads present at more than 3 m high, a small fraction of the bromeliad (especially those from *Aechmea bracteata*) was cut and all the leaves were washed in order to recover all sediments. Samples obtained were washed with drinking water through 40μm sieves and then fixed in 96% undenatured ethanol. Additionally, water samples from nearby epigeous waterbodies were collected with a standard hand plankton net (100μm mesh) and fixed in in 96% undenatured ethanol when present. Crustaceans were sorted out from samples and preserved in vials with 96% undenatured ethanol at -20°C.

### Designing primers

Due to very low success rates of COI amplification in freshwater cyclopoid species, a set of forward and reverse primers were designed. To do so, 49 COI sequences (683–707 bp) from freshwater cyclopoids consisting of eight genera (*Acanthocyclops* Kiefer, 1927, *Mesocyclops* Sars G.O., 1914, *Tropocyclops* Kiefer, 1927, *Macrocyclops* Claus, 1893, *Thermocyclops* Kiefer, 1927, *Megacyclops* Kiefer, 1927, *Cyclops* Müller O. F., 1785 and *Eucyclops* Claus, 1893) retrieved from NCBI were aligned together with our own unpublished datasets using MAFFT v7.017 [29]. Forward and reverse primers were designed for the conserved region at 5`and 3`ends of COI, resulting in a PCR product of 634 bp. Forward primer Cyclop-F (GGAACTTTGTATTTATTAGCTGGTGC, 24 bp and Tm = 56°C) and reverse primer Cyclop-R (GGTCTCCCCCTCCTCTAGG, 19 bp and Tm = 59°C) were tested in silico using Geneious 9.1.8 (Biomatters development team) for successful primer binding sites. The designed primers were synthesized in Biomers.net and used for this study.

### Sequencing and genetic analysis

DNA extractions from 255 specimens all from the second year of sampling were carried out using 30 μl Chelex (InstaGene Matrix, Bio-Rad) according to the protocol [30]. Supernatant was separated and directly used as DNA template for PCR. The mtCOI gene was amplified using different sets of primers depending on the group: for *Halicyclops* Cyclop_F and Cyclop_R primers (designed in this study), for *Elpidium* LCO-1490 and HCO-2198 [31], and for *Callistocypris* sp., *Epactophanes richardi*, *Phyllognathopus viguieri* clade 1, *Phyllognathopus viguieri* clade 2 the universal primers coxf and coxr1 were used [32]. Amplifications were performed using AccuStart GelTrack PCR SuperMix (ThermoFisher Scientific) in a 25 μl volume containing 9.5 μl H_2_O, 12.5 μl PCR Master Mix, 0.5μl of each primer (10pmol/μL) and 2 μl of DNA template. The PCR protocol was 94°C for 3 min, 94°C for 30 s, 45°C for 45 s, and 72°C for 1 min, during 35 cycles and as final elongation 72°C for 2 min. In addition, the V1V2 hyper variable region of 18S rRNA gene (~360 bp) was also amplified using the universal primers SSU_F04 and SSU_R22 [33] from some selected specimens of the different species. Amplification of V1V2 fragment was carried out using PuReTaq Ready-To-Go PCR Beads (GEHealthcare) in 25 μl volume containing 22 μl H_2_O, 0.5 μl of each primer (10 pmol/μl) and 2 μl DNA templates. The PCR protocol was 95°C for 2 min, 94°C for 1 min, 57°C for 45 s, and 72°C for 3 min, during 40 cycles and as final elongation 72°C for 5 min. To further investigate genetic diversity between two morpho-species (clades) of *Phyllognathopus viguieri* (109 specimens), the complete 18S rRNA gene (~1800 bp) has been amplified using the universal primers 18SE-F [34] and 18SL-R [35]. In addition to those, F1, CF2, CR1 and R2 internal primers [36] were used as intermediate primers for sequencing the complete forward and reverse strands. The amplification was performed using AccuStart GelTrack PCR SuperMix (for details see above). The PCR protocol was 94°C for 3 min, 94°C for 30 s, 47°C for 1 min, and 72°C for 1 min, during 35 cycles and as final elongation 72°C for 1 min.

PCR products of all markers were checked by electrophoresis and successful products were purified using ExoSap-IT PCR Product Cleanup (Thermo Fisher Scientific). Sequencing was carried out by Macrogen (Amsterdam, Netherlands). Forward and reverse strands from each specimen and fragment were assembled, edited and checked for the correct amino acid translation frame using Geneious 9.1.7 (created by Biomatters; available from http://www.geneious.com). All sequences were searched in GenBank using BLASTN [37]. Edited DNA sequences for each marker were aligned separately using MAFFT v7.017 with the G-INS-i algorithm [29], and further edited manually to exclude ambiguous regions. GenBank accession numbers for all specimens and markers are provided in S2 Appendix.

A Bayesian analysis was conducted using BEAST (Bayesian Evolutionary Analysis Sampling Trees version v1.8.3) for each fragment using the GTR nucleotide substitution model, “Strict” clock, and Yule speciation tree prior. Posterior probabilities were estimated using 10,000,000 Markov chain Monte Carlo (MCMC) generations with sample frequency of 1000 trees [38]. A maximum likelihood tree with median branch lengths was selected, discarding the 25% of the trees using TreeAnnotator v.1.8.3 [38]. The single threshold General Mixed Yule Coalescent model (GMYC) [39, 40] was used as species delimitation method. The GMYC method implemented in the R package “splits” was applied to the COI, SSU and the concatenated ultrametric trees calculated with BEAST v1.8.3 [38]. For *Phyllognathopus viguieri* clade 1 and *Callistocypris* sp. nucleotide diversity (Π) and neutrality test using Tajima’s D [41] were calculated with PopART [42]. A statistical parsimony method was used to construct a Minimum Spanning haplotype network of the mtCOI gene with PopART (http://popart.otago.ac.nz) for the widely distributed *Phyllognathopus viguieri* clade 1 (127 specimens) and *Callistocypris* sp (79 specimens). Nonparametric Mantel tests were used to calculate the correlation between input distance matrices of samples. Pairwise correlations were calculated between the genetic divergence of individual mtCOI sequences and the geographic distances. The Euclidean metric was used to compare geographic and genetic pairwise distances. In order to compare genetic variation in mtCOI among individual *P*. *viguieri*, as well as *Callistocypris* sp. sampled from different localities, p-distance genetic divergence were calculated using MEGA7: Molecular Evolutionary Genetics Analysis v. 7.0 [43].

### Statistical analysis

In order to determine which species are associated to a particular bromeliad species/biosphere reserve, the IndVal index using the multipatt function implemented in the “indicspecies” R package [44] was calculated. For community analysis, data were standardized using the Hellinger transformation with the function decostand implemented in the “vegan” R package [45]. The similarity in community structure between bromeliad species and reserves was further analyzed with standard community analysis tools, pairwise.adonis [46] was computed in R, using Euclidean distance as the similarity method with BH-adjusted p-values. Multivariate dispersion between bromeliad species/reserves was computed using the function betadisper implemented in the “vegan” package for R [45]. The statistical software R 3.5.1 (R Core Team, 2018) was used to perform all statistical analyses.

## Results

Sediments and water collected from 238 bromeliads belonging to five different species, i.e., *Aechmaea bracteata*, *Aechmea* sp., *Tillandsia dasyliriifolia*, *T*. *fasciculata* and *Tillandsia* sp. “mangrove bromeliad”were analyzed. Fifty-five percent of bromeliads (131 plants) were colonized by copepods, ostracods and, from one plant (*A*. *bracteata*) two individuals of branchiopod were recorded, *Simocephalus mixtus* Sars, 1903. In addition to crustaceans, other groups have been found in bromeliads sampled but they have not been included in our analyses: Arachnida (spiders, acari and pseudoscorpions), Nematoda, Rotifera (Bdelloidea and Monogononta), Collembola, Tardigrada and several insect larvae.

**Fig 2:**
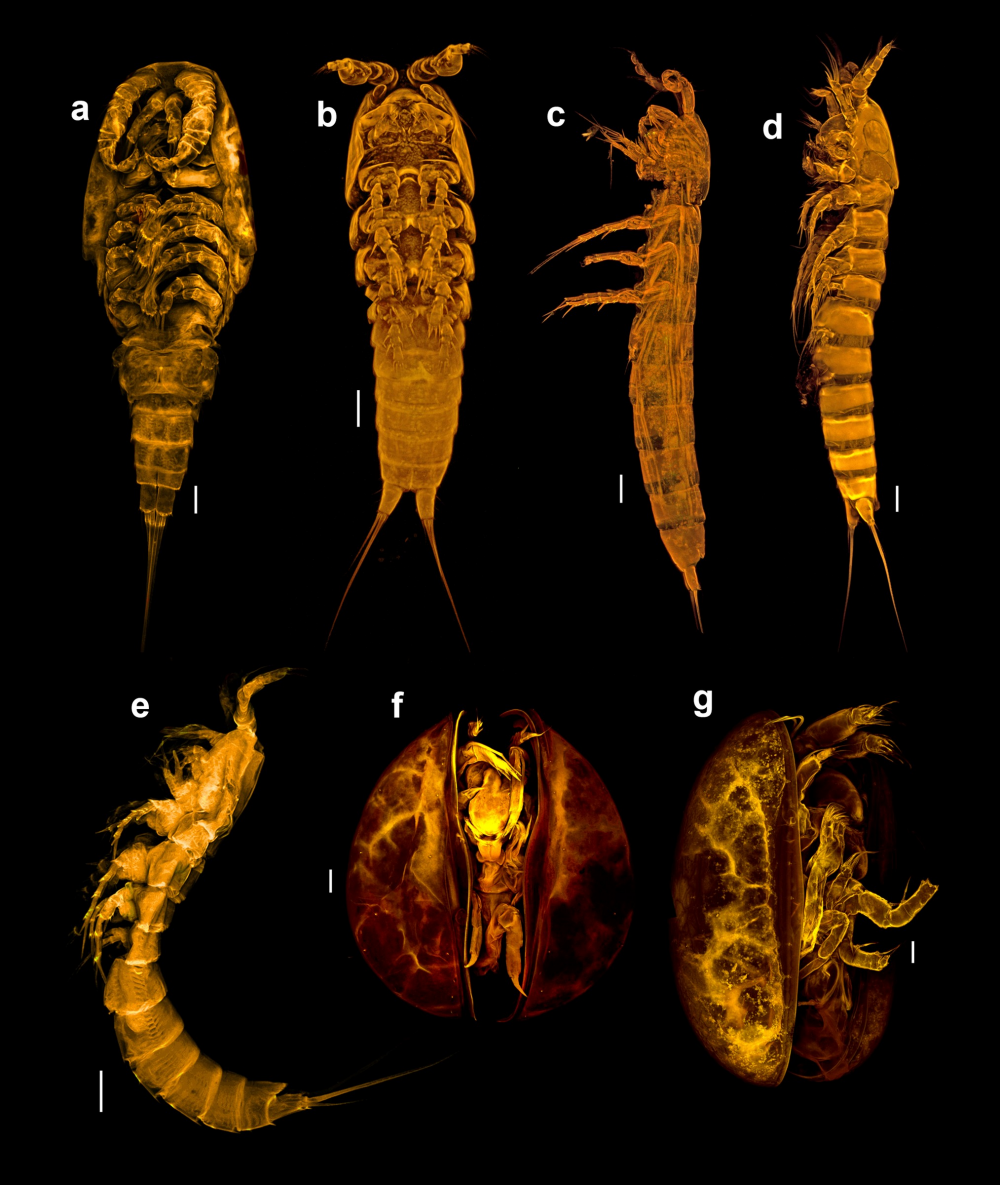
CLSM images of the bromeliad crustacean taxa. a) *Halicyclops* sp. 2, ventral; b) *Epactophanes richardi*, ventral; c) *Remaneicaris siankaan*, lateral; d), *Phyllognathopus viguieri* clade 1, lateral; e) *Phyllognathopus viguieri* clade 2, lateral; f) *Elpidium* sp.; g) *Callistocypris* sp. Scale bars = 25μm.

Sixteen copepods, three ostracods and one branchiopod species were recorded during this study; however, only seven species are considered as true bromeliad inhabitants (Fig 2) because of the presence of several individuals including all life stages, i.e., males, females and larvae. The other 13 species were recorded by the presence of only one individual per bromeliad. The number of specimens within single bromeliads varied from few to hundreds of individuals. All species, with exception of *Phyllognathopus viguieri* which was considered here as *P*. *viguieri* s. sl. (Maupas, 1892) and *E*. *richardi* Mrázek, 1893, are new to science but their descriptions will be presented elsewhere (*R*. *siankaan* was described in [47]). *Phyllognathopus viguieri* clade 1, *P*.*viguieri* clade 2, *E*. *richardi*, *R*. *siankaan* and *Halicyclops* sp. 2 are the five copepod species considered as true bromeliad inhabitants, while for ostracods two species were included in this category; *Callistocypris* sp. and *Elpidium* sp.

The most successful bromeliad colonizer was *Callistocypris* sp. present in 30% of the bromeliads analyzed, followed by *P*. *viguieri* clade 1 (20%), *E*. *richardi* (7%), *Elpidium* sp. (4%), *R*. *siankaan* (3%), *Halicyclops* sp. 2 (3.8%) and *P*. *viguieri* clade 2 (1.2%). A complete list of crustacean species and occupied bromeliad species is provided in Table 1. Three different crustacean species with the highest species richness were found in a single bromeliad; however, only two bromeliads contained such diversity. The great majority (101 bromeliads) was inhabited by a single crustacean species and twenty-eight bromeliads contained two species of crustaceans, most of them inhabited by one copepod and one ostracod species. Additionally, a list of copepods found in nearby epigeous waterbodies is provided in Table 2. The only species that occupied both habitats was *Remaneicaris siankaan*, as all others were either common to bromeliads or to epigeous waterbodies.

**Table 1:** List of aquatic crustaceans recorded from bromeliads.

Family	Species	Bromeliad
**COPEPODA**		
**Harpacticoida**		
Phyllognathopodidae	*Phyllognathopus viguieri* clade 1*	Ab(32), Td (5), Tf (2), Tsp. (3)
	*Phyllognathopus viguieri* clade 2*	Asp. (3)
Canthocamptidae	Epactophanes richardi*	Ab (4), Td (4), Tf (7),
Parastenocarididae	Remaneicaris siankaan*	Td (7)
Ameiridae	*Nitokra lacustris*	Tsp. (1)
	*Nitokra spinipes*	Tsp. (1)
Miraciidae	*Schizopera* sp.	Tsp. (1)
**Cyclopoida**		
Cyclopidae	*Apocyclops panamensis*	Td (1)
	*Thermocyclops inversus*	Ab (1)
	*Tropocyclops prasinus aztequei*	Ab (1)
	*Microcyclops rubellus*	Tsp. (1)
	*Microcyclops* sp.	Tf (1)
	*Neutrocyclops brevifurca*	Td (1)
	*Macrocyclops albidus*	Ab (1)
Halicyclopidae	*Halicyclops* sp.2*	Tsp. (5)
**Calanoida**		
Diaptomidae	*Mastigodiaptomus* sp.	Ab (1)
**OSTRACODA**		
**Podocopida**		
Cyprididae	*Callistocypris* sp.*	Ab (34), Td (10), Tf (23), Tsp. (1)
Limnocytheridae	*Elpidium* sp.*	Ab (9)
Candonidae	*Caaporacandona* sp.	Ab (2)
**BRANCHIOPODA**		
**Anomopoda**		
Daphniidae	*Simocephalus mixtus*	Ab (1)

Species marked with (*) represent the species considered as true bromeliad colonizers. Bromeliad species were abbreviated as follows: (Ab) *Aechmea bracteata*, (Td) *Tillandsia dasyliriifolia*, (Tf) *Tillandsia fasciculata*, (Tsp.) *Tillandsia* sp.–mangrove bromeliad- and (Asp.) *Aechmea* sp. Numbers in () represent the number of bromeliads colonized *per* species.

**Table 2:** List of copepod species found in epigeous waterbodies nearby bromeliad collecting sites.

Locality	Reserve	Habitat	Species
Ramonal Km 27	Calakmul	Aguada	*Ectocyclops rubescens*, *Mesocyclops brasilianus*, *Mesocyclops longisetus*, *Microcyclops ceibaensis*, *Microcyclops echinatus*, *Tropocyclops prasinus aztequei*
			*Thermocyclops inversus*
Aguada límite de la Reserva	Sian ka´an	Aguada	*Mastigodiaptomus siankaanensis*, *Acanthocyclops* sp. nov., *Mesocyclops reidae*, *Microcyclops ceibaensis*, *Microcyclops rubellus*, *Neutrocyclops brevifurca*
			*Thermocyclops inversus*, ***Remaneicaris siankaan***
Arroyo Aguada Grande	Calakmul	Creek	*Mastigodiaptomus reidae*, *Mesocyclops pescei*, *Eucyclops prionophorus*
			*Acanthocyclops* sp. nov.
Arroyo Calakmul	Calakmul	Creek	*Mastigodiaptomus reidae*, *Acanthocyclops* sp. nov., *Thermocyclops inversus*
Dos Naciones	Calakmul	Creek	*Mesocyclops edax*, *Thermocyclops inversus*
Banco Material Camino Pulticub	Sian Ka´an	Pond	*Microcyclops ceibaensis*
Cenote Aguada 1	Sian ka´an	Cenote	*Macrocyclops albidus*, *Microcyclops ceibaensis*, *Paracyclops chiltoni*, *Thermocyclops inversus*, *Tropocyclops extensus*
Cenote Domin	Sian ka´an	Cenote	*Ectocyclops rubescens*, *Eucyclops festivus*, *Macrocyclops albidus*, *Microcyclops ceibaensis*, *Microcyclops rubellus*, *Thermocyclops inversus*, *Tropocyclops prasinus mexicanus*
Cruce Villahermosa	Calakmul	Pond	*Thermocyclops inversus*
Laguna Mosquitero	Sian ka´an	Lagoon	*Acanthocyclops* sp. nov., *Apocyclops panamensis*, *Mesocyclops chaci*, *Mesocyclops reidae*, *Microcyclops rubellus*
Playón	Sian ka´an	Wetland-Mangrove	*Apocyclops panamensis*, *Halicyclops* sp. 1, *Microcyclops rubellus*, *Microcyclops* sp. 1, *Nitokra lacustris*, *Nitokra spinipes*
Poyanca Bejuco Muk	Calakmul	Well	*Mesocyclops longisetus*, *Microcyclops ceibaensis*, *Microcyclops echinatus*, *Neutrocyclops brevifurca*, *Thermocyclops inversus*
Pozo 1 Rancho Alejandro	Sian ka´an	Well	*Macrocyclops albidus*, *Mesocyclops chaci*, *Mesocyclops reidae*
Pozo 2 Rancho Alejandro	Sian ka´an	Well	*Mesocyclops reidae*
Campamento Militar Villahermosa	Calakmul	Well	*Mesocyclops chaci*
Savannah 2	Sian ka´an	Wetland	*Diacyclops* sp. 1, *Macrocyclops albidus*, *Microcyclops echinatus*, *Acanthocyclops* sp. nov., *Mesocyclops chaci*, *Mesocyclops reidae*, *Paracyclops chiltoni*, *Mastigodiaptomus siankaanensis*, ***Remaneicaris siankaan***
Pulticub km 44	Sian ka´an	Wetland-Mangrove	*Apocyclops panamensis*, *Schizopera* sp.
Savannah Km 10	Sian ka´an	Wetland	***Remaneicaris siankaan***
Vigía Chico	Sian ka´an	Wetland	*Mastigodiaptomus siankaanensis*, *Acanthocyclops* sp. nov., *Microcyclops ceibaensis*
			*Microcyclops echinatus*, *Mesocyclops reidae*, ***Remaneicaris siankaan***

Different degrees of association between crustaceans and bromeliad species/reserves were uncovered with an indicator species analysis (Table 3). *Aechmea* sp. 1, *Tillandsia dasyliriifolia*, *Tillandsia* sp. had at least one significant relationship with a particular crustacean species. The widely distributed *Callistocypris* sp. was associated with a group formed by *Aechmea bracteata + Tillandsia dasyliriifolia + Tillandsia fasciculata*, and *P*. *viguieri* clade 1 was associated with a group formed by *Aechmea bracteata* + *Tillandsia* sp.1. For the reserves, Calakmul was associated with *Epacthophanes ricardi* and *Phyllognathopus viguieri* clade *2*, while *P*. *viguieri* clade 1 and *Callistocypris* sp. were associated with Sian ka´an Reserve.

**Table 3:** Significant groups obtained by the indicator species analysis.

Bromeliad group	Crustacean species associated	p-value
*Aechmea* sp. 1	*Phyllognathopus vigueri* 2	0.005 **
*Tillandsia dasyliriifolia*	*Remaneicaris siankaan*	0.005 **
*Tillandsia* sp. 1	*Halicyclops* sp. 2	0.005 **
*Aechmea bracteata+Tillandsia* sp. 1	*Phyllognathopus vigueri* 1	0.005 **
*Aechmea bracteata+Tillandsia dasyliriifolia+Tillandsia fasciculata*	*Callistocypris* sp.1	0.05*
Calakmul	*Epactophanes richardi*	0.005 **
	*Phyllognathopus vigueri 2*	0.010 *
Sian ka´an	*Phyllognathopus vigueri* 1	0.005 **
	*Callistocypris* sp. 1	0.010 **

Significance codes: 0

‘***’ 0.001

‘**’ 0.01

‘*’ 0.05 ‘.’ 0.1 ‘ ‘ 1

Comparisons between bromeliad/reserve species and their associated crustacean fauna was tested using pairwise analysis (PERMANOVA), Adonis in which the differences between bromeliad species (p = 0.001) and reserves (p = 0.001) were significant. A pairwise post-hoc test revealed that every pair of bromeliads and reserves were significantly different from each other (Table 4). To stress the significant differences in multivariate dispersion between bromeliad species/reserves, a betadisper analysis was performed in which a posthoc pairwise test revealed that bromeliad species differed significantly in multivariate dispersion (p = 0.001) but not reserves (p = 0.575).

**Table 4:** Results of pairwise.adonis comparing different bromeliad species and reserves.

Bromeliad/Reserve Pair	p. value	p. adjusted
*Aechmea bracteata* vs *Tillandsia fasciculata*	0.001	0.002*
*Aechmea bracteata* vs *Tillandsia dasyliriifolia*	0.008	0.008*
*Aechmea bracteata* vs *Aechmea* sp. 1	0.001	0.002 *
*Aechmea bracteata* vs *Tillandsia* sp. 1	0.001	0.002 *
*Tillandsia fasciculata* vs *Tillandsia dasyliriifolia*	0.002	0.003 *
*Tillandsia fasciculata* vs *Aechmea* sp. 1	0.002	0.003 *
*Tillandsia fasciculata* vs *Tillandsia* sp. 1	0.001	0.002*
*Tillandsia dasyliriifolia* vs *Aechmea* sp. 1	0.001	0.002 *
*Tillandsia dasyliriifolia* vs *Tillandsia* sp. 1	0.004	0.005 *
*Aechmea* sp. 1 vs *Tillandsia* sp. 1	0.008	0.001 **
Calakmul vs. Sian ka´an	0.001	0.001**

Significance codes: 0

‘***’ 0.001

‘**’ 0.01

‘*’ 0.05 ‘.’ 0.1 ‘ ‘ 1

**Fig 3:**
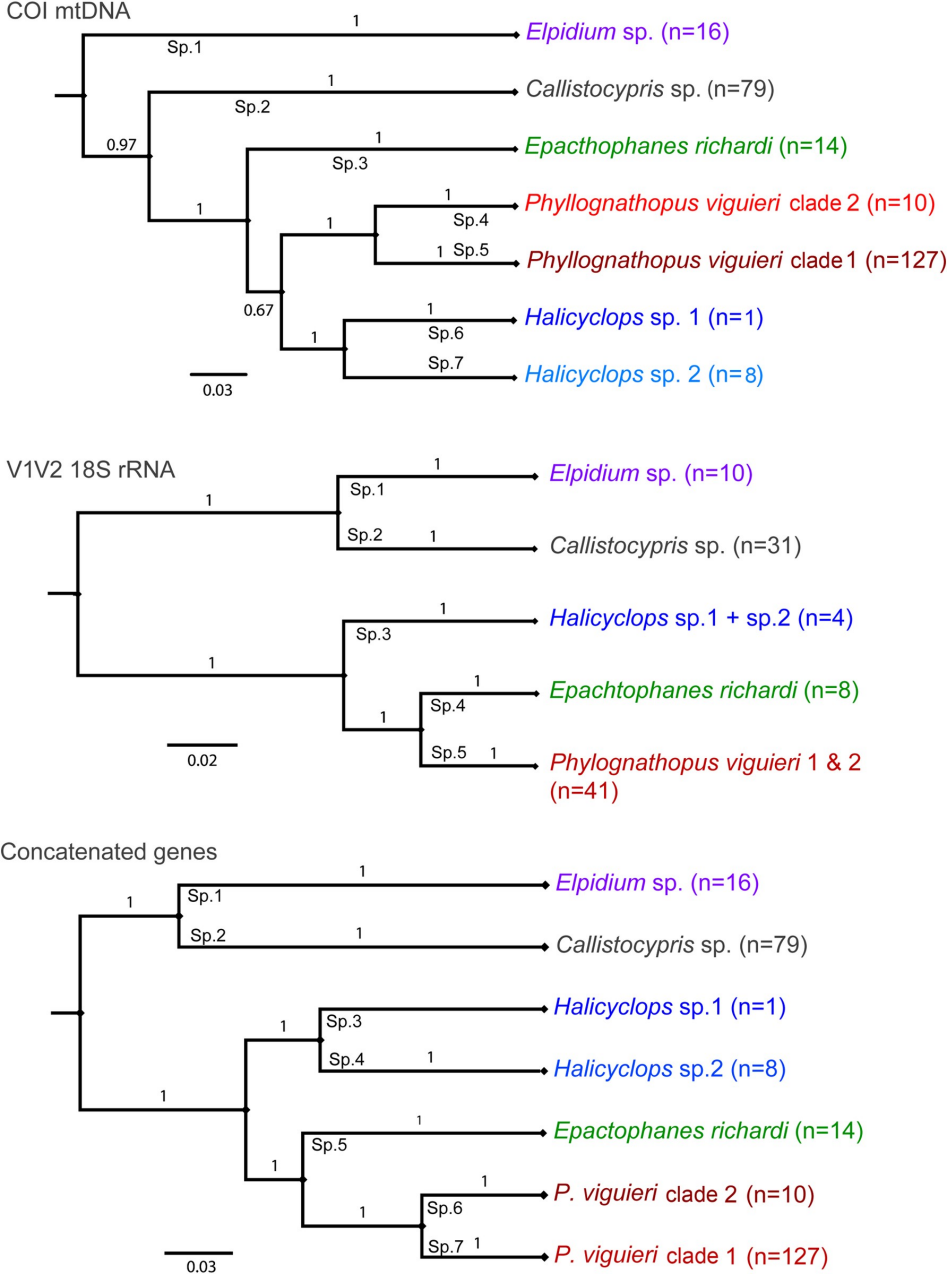
Phylograms generated by Bayesian analyses. a) COI mtDNA sequences; b) V1V2 18SrRNA; c) Concatenated alignment of both fragments. Branches are collapsed to distinct clades according to the GMYC delimited species. Values on branches are posterior probabilities. The enumeration of species (on the branches in black) represents species supported by General Mixed Yule Coalescent model (GMYC).

For all bromeliad-specific species we obtained successful sequences, with the exception of *Remaneicaris siankaan*. We included a second species of *Halicyclops* sp. 1 found in water samples taken from the same mangrove area, Playón, where the bromeliads containing *Halicyclops* sp. 2 were found. Fig 3 shows the Bayesian trees generated for COI mtDNA, 18S rRNA (V1V2), and the multigene tree from the concatenated alignment of both markers, respectively. The Generalized Mixed Yule Coalescent analysis (GMYC) identified seven species with mtCOI (Fig 3A) and 5 species with the V1V2 (Fig 3B). COI delimited 2 species for *Phyllognathopus* and *Halicyclops* each, however V1V2 did not support this clustering and recovered one species per genus. The concatenated tree of both fragments distinguished 7 species by GMYC, congruent with COI (Fig 3C).

A Mantel test was performed to analyze the correlation between COI genetic divergence and geographic distance for the ostracod *Callistocypris sp*. and both clades of *Phyllognathopus* Mrázek, 1893. For *Callistocypris* sp. no correlation was found between genetic distance and geographic distance (R = 0.02159, p = 0.2066) while the opposite was revealed for *Phyllognathopus* clades where a significant association between genetic distance and geographical distance indicated fine-scale genetic population structure (R = 0.7302, p = 0.001).

Intra and inter-specific COI variabilities are provided in Table 5 for genetic diversity within and between individuals in our study. For individuals of *P*. *viguieri* clade 1, the minimum intra-genetic COI variabilities (p.distance = 0%) were recovered for the Savannah 2 population, whereas Vigía Chico showed the highest genetic divergence, 0.55%, among all other localities (Table 5). Between locations, Playón and Savannah Playón showed the highest genetic similarities for the *P*. *viguieri* clade 1 (p.distance = 0.15%), while individuals from Vigía Chico and Camino Pemex had the highest genetic distances among all others (p.distance = 1.25%). Between the two *P*. *viguieri* clades (clade 1 only from Sian Ka´an reserve and clade 2 only Calakmul), there were COI genetic differences of 17.14%– 17.43% which further indicates the presence of two species, each distributed in one reserve. The minimum (0%) and maximum (0.55%) COI intra-genetic diversity was recovered for *Callistocypris* from each locality. Individuals from Cruce Villahermosa, Calakmul were highly genetically similar to the Aguada Limite Reserva from Sian Ka´an (p.distance = 0.24%), while Camino Pemex (Sian Ka´an) showed the maximum genetic distances in comparison to Arroyo Aguda Grande (Calakmul, 0.78%).

**Table 5:** COI genetic inter and intra specific variabilities (p.distance) among different populations (localities).

**a) *Phyllognathopus viguieri* clade 1 & 2**									
	CP	VC	Pl	SP	Pu	Sa 2	**Ca (P.v.2)**		
Camino_Pemex (S)	0.06%								
Vigía_Chico (S)	1.25%	0.55%							
Playón (S)	0.72%	0.70%	0.21%						
Savannah_Playón (S)	0.63%	0.67%	0.15%	0.07%					
Pulticub (S)	1.17%	0.90%	0.56%	0.59%	0.44%				
Savannah_2(S)	1.22%	0.53%	0.62%	0.62%	0.65%	0%			
**Calakmul (*P*. *viguieri* 2)**	**17.43%**	**17.21%**	**17.15%**	**17.14%**	**17.14%**	**17.39%**	**0.11%**		
**b) *Callistocypris* sp.**									
	Sa 10	ALR	Ra 27	CV	AAG	Pl	VC	CP	AQR
Savannah_km_10 (S)	0.54%								
Aguada_Límite_Reserva (S)	0.58%	0.10%							
Ramonal_Km_27 (C)	0.64%	0.26%	0.34%						
Cruce Villahermosa (C)	0.63%	0.24%	0.29%	0.33%					
Arroyo_Aguda_Grande (C)	0.69%	0.29%	0.38%	0.37%	0.08%				
Playón (S)	0.67%	0.25%	0.36%	0.34%	0.38%	0%			
Vigía_Chico (S)	0.44%	0.25%	0.36%	0.34%	0.38%	0.34%	0%		
Camino_Pemex (S)	0.43%	0.66%	0.76%	0.75%	0.78%	0.74%	0.40%	0.09%	
Andres_Quintana_Roo (S)	0.45%	0.64%	0.68%	0.68%	0.76%	0.73%	0.41%	0.26%	0.44%

**a)** *Phyllognathopus viguieri* clade 1 and 2, b) *Callistocypris* sp. Specimens of *P*. *viguieri* clade 2 are only presented in Calakmul reserve and are highlighted in the table. (C) = Calakmul, (S) = Sian ka´an.

**Fig 4:**
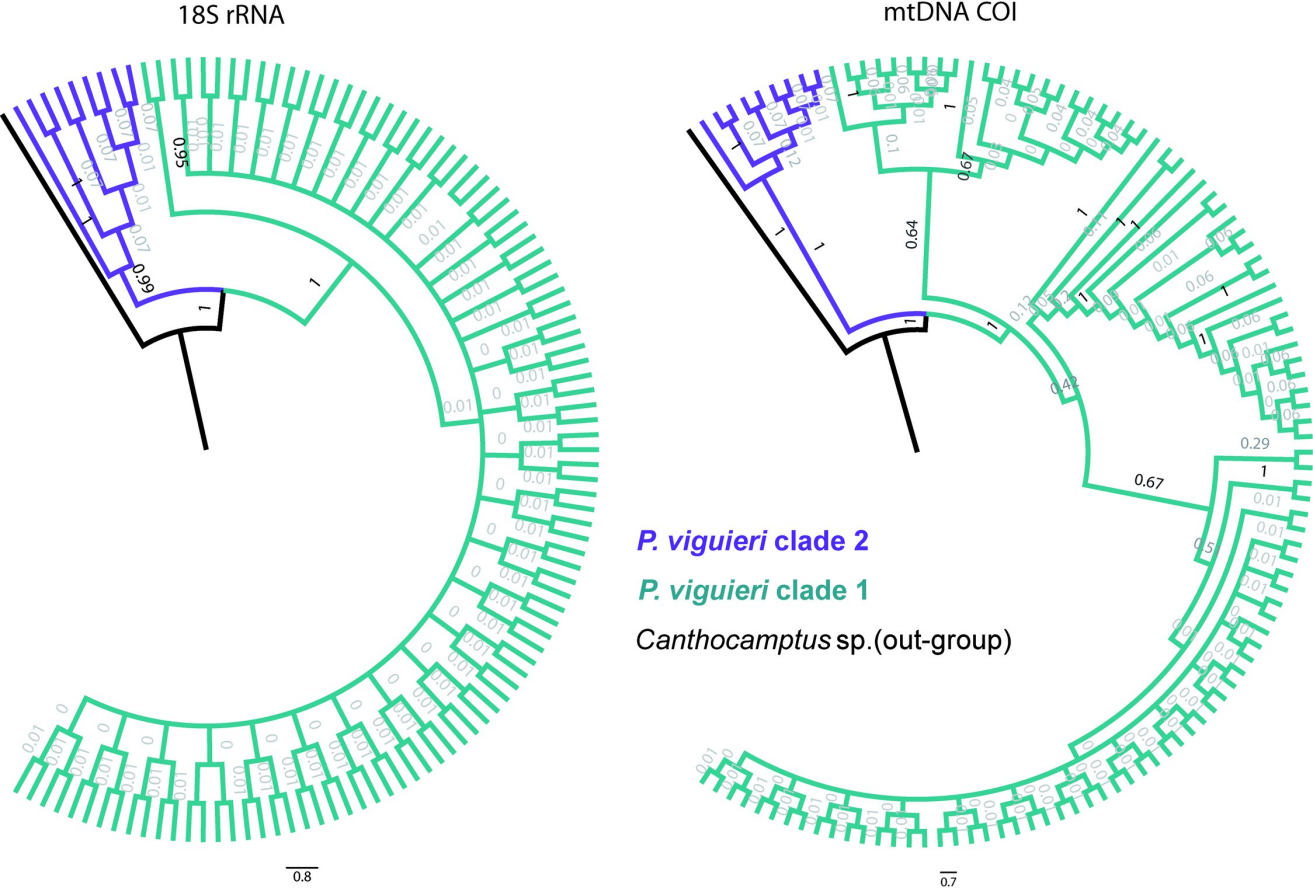
Circle-shaped phylogenetic trees generated by Bayesian analyses. a) 18S rRNA; b) COI mtDNA. The distinct *Phyllognathopus* clades are represented by different colors; green color is given to *Phyllognathopus viguieri clade 1* andviolet represents *Phyllognathopus viguieri clade 2*. Values on branches are posterior probabilities in which the probabilities lower than 0.60% are shown in light gray.

In order to test the accuracy and robustness of the 2 *Phyllognathopus* species which have delimited only by COI and not the V1V2 gene fragment, the complete 18S rRNA (~1800 bp) was sequenced from 109 individuals of *Phyllognathopus*. The trees generated by Bayesian analyses from the 18S rRNA gene resulted in a similar topology to that from mtCOI showing a robust separation (posterior probabilities = 1) between the two clades of *P*. *viguieri* clade 1 and *P*. *viguieri* clade 2 (Fig 4).

**Fig 5:**
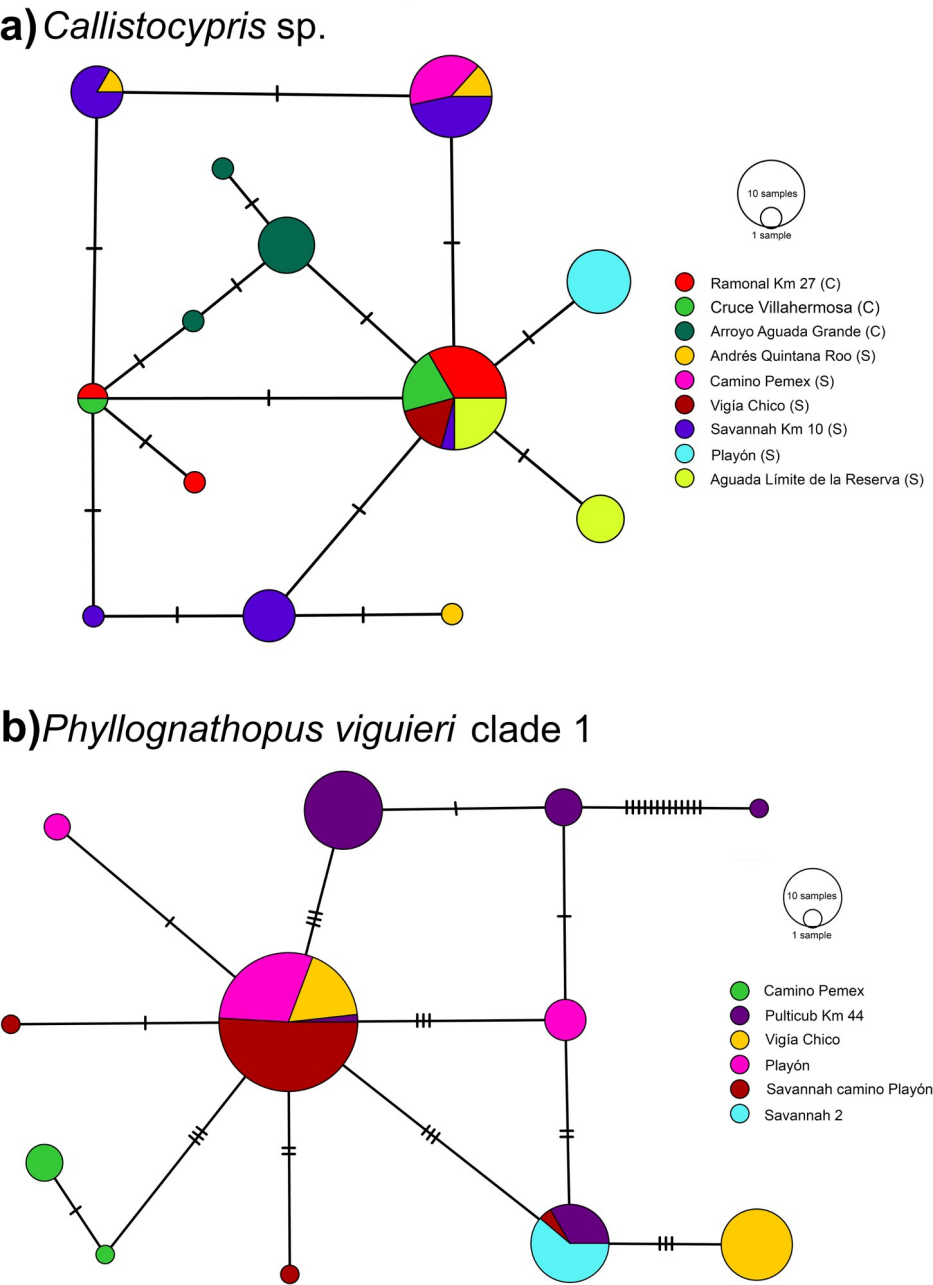
mtCOI haplotype networks. Cross lines on the branches represent biallelic mutational numbers. The size of circles is proportional to haplotype frequency. Color indicates haplotype location. a) *Callistocypris* sp., letters within () indicate the Reserve where locality is found: (C) = Calakmul, (S) = Sian ka´an. b) *Phyllognathopus viguieri* clade 1, all localities are inside Sian ka´an Biosphere Reserve.

Population analyses of *Callistocypris* sp. revealed 8 polymorphic sites with nucleotide diversity of Π = 0.0035 from 13 different haplotypes (Fig 5A). The most common haplotype was found in both reserves, 5 haplotypes were found exclusively in Calakmul, and 7 in Sian ka´an, which yielded a fixation index, F_ST_ = 0.5094; *P* < 0.01. The minimum spanning network of *P*. *viguieri* 1 revealed 28 polymorphic sites (Π = 0.005) and 12 different haplotypes (Fig 5B) in which F_ST_ = 0.4938 (*P* < 0.01) among different populations from the Sian Ka´an Biosphere Reserve.

## Discussion

This study is the first extensive survey of micro-crustaceans associated with bromeliads in Mexico, and the first survey on copepod phytotelms using multi-marker genetic data worldwide. Until now, twelve species of micro-crustaceans have been reported inhabiting Mexican bromeliads; however, all these records belong to species that are widely distributed in epigeous water bodies throughout the country [10, 19, 20]. The only exception is *Olmeccyclops veracruzanus* known from the bromeliad *Tillandsia heterophylla* in Veracruz [19]. From the twenty species of crustaceans found during this study only *Thermocyclops inversus* (Kiefer, 1936) was previously recorded inhabiting bromeliads in Mexico, thus the rest of the species represent new records associated with phytotelmata. One species of the genus *Mastigodiaptomus* Light, 1939 represent the first member of the order Calanoida Sars G.O. 1903 ever recorded in phytotelmata worldwide. However, this finding should be considered with caution because only one individual was found in sediments collected from one *Aechmaea bracteata* at Arroyo Aguada Grande.

Seven of the twenty crustacean species collected during this study are considered true bromeliad inhabitants, i.e., *Phyllognathopus viguieri* clade 1, *P*.*viguieri* clade 2, *E*. *richardi*, *R*. *siankaan*, *Halicyclops* sp. 2, *Callistocypris* sp. and *Elpidium* sp. With the exception of *R*. *siankaan*, none of these species were reported in epigeous water bodies sampled during both sampling campaigns (see Table 2). This strongly indicates that bromeliad crustaceans are specialized to such environments as previously reported for ostracods in Jamaican bromeliads [48]. Additional evidence of the specialization of bromeliad crustaceans to their habitat was suggested by the indicator species analysis, where statistical significance of the association between all crustacean species and a site group (in our case a bromeliad species) was recovered (see Table 3).

Fifty-five percent of the bromeliads surveyed were colonized by crustaceans, where copepods and ostracods colonized 32% of the plants. Higher success of crustaceans colonizing bromeliads has been previously recorded in other regions in the Americas, especially in Brazil and Jamaica [12, 14, 28, 48]. For Jamaican bromeliads, ca. 81% of bromeliads surveyed were colonized by ostracods belonging to the genera *Elpidium*, *Candonopsis* Vavra, 1891 and an undescribed genus [48]. In a different study on Jamaican bromeliads, 90% of the plants were occupied by ostracods identified as *Cypridae* sp. and *Metacypris* sp. [14]. As well, the same percentage of plants was occupied by the copepods *Defayeicyclops jamaicensis* (Reid & Janetzy, 1996) and *Ectocyclops phaleratus* (Koch, 1838) [14]. In a study concerning phoretic dispersal agents of bromeliad ostracods in Brazil, a high percentage of bromeliads were also colonized (89%) by members of the genus *Elpidium* [12]. The same percentage of plants colonized by *Elpidium* (89%) was reported in a study comparing the richness and faunal composition in tanks exposed to sun-versus shaded areas [13]. Jocque et al. [28] reported 59% of the bromeliads surveyed in Cusco National Park in Honduras were occupied by ostracods but no other information about the genera or species was provided. Additional records of crustaceans occupying bromeliads can be found in several papers, however the percentages of colonized plants are not included [11, 49–54].

The prevalence of endemic ostracods identified by genetic methods has been previously studied in Jamaican bromeliads [48], where 11 independent lineages, 10 being exclusive to bromeliads, have been found (two for *Candonopsis* and 9 for *Elpidium*). In these studies, nine different *Elpidium* species showed diagnostic genetic attributes together with differences in male morphology including the distal lobe of the hemipenes. In six cases of sympatry, i.e., co-occurring in a single bromeliad, there was no evidence of hybridization (D = 0.36–0.52) found in an allozyme variation analysis. Furthermore, these authors estimated the genetic divergence of COI Restriction Fragment Length Polymorphism (RFLP) to confirm that different species, and even conspecific populations, were highly genetically divergent with average Nei´s genetic distance = 8.4%. Unfortunately, comparison of our genetic data and the above results [48] is not possible due to the different methodologies used to assess species delimitation, and no COI sequences from their work are available in public databases as BOLD or NCBI.

During our study, members of the genus *Elpidium* sp. presented a restricted distribution being present only at two localities in Sian ka´an Biosphere reserve; where only one haplotype has been found. For *Callistocypris* sp. one widely distributed species with 13 different haplotypes was found in different localities of both Reserves, with a COI genetic p.distance = 0.54% among populations, and Mantel Tests revealed no correlation between genetic distance and geographic distance. For instance, the highest genetic similarity among *Callistocypris* populations has been found between localities 273.15 km apart from each other (Cruce Villahermosa and Aguada límite de la Reserva). As well, the maximum genetic distance was found in localities 233.79 km apart (Arroyo Aguada Grande and Camino Pemex. This pattern suggests great dispersal capacities over long distances for the species, contrary to what has been formulated for Jamaican ostracods [48].

The opposite situation has been revealed for *Phyllognathopus* species where a significant association between genetic distance and geographical distance indicated fine-scale genetic population structure. At first glance, the morphology of *Phyllognathopus viguieri* clade 1 and *P*. *viguieri* clade 2 were recognized as the same lineage, but molecular evidence of both mitochondrial and nuclear genes including complete ribosomal 18S sequences showed that they represent two independent lineages with a mtCOI p.distance of 17.43% with disjunctive geographic distributions. Individuals from the first clade were found in several bromeliads sampled from different localities in the Sian ka´an Reserve while the second was only found in one locality, Dos Naciones in the Calakmul Reserve, which was the southernmost sampling site during this study. *Phyllognathopus viguieri* clade 1 was represented by 12 haplotypes distributed in 6 localities in Sian ka´an (Fig 5B) while two haplotypes of *Phyllognathopus viguieri* clade 2 were found in Calakmul.

The island nature of bromeliad habitats and their temporality are limiting factors for many of the organisms inhabiting them. Therefore, bromeliad inhabitants possess anatomical features and/or life history traits that allow them to invade and survive fragmented and ephemeral freshwater microcosms [12, 48, 55]. A key factor in the success of these animals is their mode and rate of dispersal, a necessary component of their demographic and evolutionary dynamics of species populations and a significant step in the colonization process [55]. During this study, no vertebrate/invertebrate fauna were checked for crustacean attachment, however different lizards, frogs, scorpions, spiders and hexapods (ants, wasps, coleopteran, etc.) were observed when sediments were removed from the plants suggesting the possibility that crustaceans, especially ostracods, could move among plants using these animals as vectors (NFM-S, pers. obs.). Several hypotheses about the mode and rate of dispersal used by bromeliad crustaceans, especially for ostracods, have been proposed. Among them are passive dispersal, i.e., passive transfer between new and old bromeliad ramets and transfer through rain water and wind, phoretic behavior, and active dispersal are the most accepted [12–14, 48, 49, 52, 55]. Several studies about phoretic behavior of ostracods, especially of the genus *Elpidium*, have been performed since 1960´s, confirming that such crustaceans can move among bromeliads attached to the skin of amphibians and reptiles. It has been even suggested that ostracods and annelids living in bromeliads have preferences for attaching themselves to specific vectors [49]. Additionally it has been shown that ostracods can survive unharmed in the digestion track of different vertebrates that visit the bromeliads to drink water or use the plants as aggregation, hiding, or resting sites [10, 12, 13, 49, 55].

The behavior of some copepod species suggests that they may climb actively into the plants [56]. For instance, the harpacticoid *Bryocamptus pygmaeus* (Sars G.O., 1863) and the cyclopoids *Paracyclops fimbriatus* (Fischer, 1853), *P*. *affinis* (Sars G.O., 1863) and *Ectocyclops phaleratus* can live in a film of water and tend to climb the walls of glass containers out of the water [56]. To our knowledge, there are no reports of copepods attached to frogs, lizards or other vertebrates sampled in different studies [12, 13, 49, 52, 55]. For instance, in the study made by Araujo et al. [55] no copepods were observed being attached or adhered to frogs, although they were present in the water held by the bromeliads were anurans were sampled, suggesting a different type of dispersal than these observed for ostracods.

There is only one known study about colonization rates of bromeliad copepods where *Defayeicyclops jamaicensis* rapid colonized and was common in phytotelmata, with a rate of spread comparable to that of the flying insects, e.g., chironomids and culicids [14]. Active colonization was suggested as the most reliable mechanism used by copepods, where phoresy and wind transport of resting eggs or other disseminules was not documented in the fauna visiting bromeliads neither in the plastic jars placed at the study site. However the question of the means used by copepods to colonize bromeliads remains open until more experimental work can be performed.

It is well known that a substantial fraction of the freshwater available in neotropical forests is impounded within the rosettes of bromeliads that form aquatic islands within terrestrial landscapes providing microhabitats for aquatic organisms where ponds and lakes are naturally scarce [57]. The ecological role and the multiple ecological services provided by bromeliad plants, as well their use as model systems to answer numerous ecological and evolutionary questions have been recently highlighted [11, 58]. This study stresses, once more, the need for study of the meiofauna of phytotelms, that could be used as an indicator of local diversity in a changing world where habitat fragmentation, disturbance and climate change are among the most important constraints for conservation and management of neotropical forests.

## Supporting information

S1 Appendix Sampling localities in Calakmul and Sian ka´an Biosphere Reserves. (PDF)

S2 Appendix GenBank accession numbers for all specimens and markers sequenced during this study. (XLSX)
