# Supplementary material for: Copepods and ostracods associated with bromeliads in the Yucatán Peninsula, Mexico
Source: PLoS One. 2021 Mar 18;16(3):e0248863. doi: 10.1371/journal.pone.0248863 (PMC7971893; doi:10.1371/journal.pone.0248863)
Supplement: S1 Appendix — (PDF) [file pone.0248863.s001.pdf]

S1 Appendix. Sampling localities in Calakmul and Sian ka´an Biosphere Reserves.

Locality	Date	Reserve	Latitude N	Longitude W
Savannah El Mirador	19. Sep 14	Sian Ka´an	19.769	87.742
Vigía Chico	20. Sep 14	Sian Ka´an	19.784	87.610
Savannah 2	21. Sep 14	Sian Ka´an	19.799	87.700
Cenote Savannah 1	22. Sep 14	Sian Ka´an	19.763	87.759
Aguada límite de la Reserva	23. Sep 14	Sian Ka´an	19.709	87.828
Cruce Villahermosa	27. Sep 14	Calakmul	18.186	89.746
Poyanca Guiro	27. Sep 14	Calakmul	18.170	89.772
Poyanca Bejuco Muk	27. Sep 14	Calakmul	18.205	89.752
Arroyo Calakmul	28. Sep 14	Calakmul	18.123	89.790
Aguada Grande (Helipuerto)	28. Sep 14	Calakmul	18.124	89.818
Arroyo Aguada Grande	28. Sep 14	Calakmul	18.123	89.815
Aguada Zona Arqueológica	28. Sep 14	Calakmul	18.109	89.806
Bajo Km 29	29. Sep 14	Calakmul	18.304	89.849
Ramonal Km 27	29. Sep 14	Calakmul	18.316	89.857
Ramonal Km 27	15. Sep 15	Calakmul	18.316	89.857
Bajo Campamento Militar Villahermosa	16. Sep 15	Calakmul	18.176	89.744
Cruce Villahermosa	17. Sep 15	Calakmul	18.011	89.713
Dos Naciones	18. Sep 15	Calakmul	17.974	89.359
Cerro El águila	19. Sep 15	Calakmul	18.327	89.952
Conhuas-Calakmul	19. Sep 15	Calakmul	18.329	89.909
Arroyo Aguada Grande	20. Sep 15	Calakmul	18.123	89.815
Bajo camino Oxpeh-muul	21. Sep 15	Calakmul	18.122	89.816
Oxpeh-muul	21. Sep 15	Calakmul	18.315	89.779
Bonfil Oeste	22. Sep 15	Calakmul	18.315	89.779
Andrés Quintana Roo	28. Sep 15	Sian Ka´an	18.060	89.719
Cenote Polvora	29. Sep 15	Sian Ka´an	19.416	87.899
Cenote Darwin	29. Sep 15	Sian Ka´an	19.422	87.937
Camino Pemex	30. Sep 15	Sian Ka´an	19.283	87.960
Laguna Mosquitero	1. Oct 15	Sian Ka´an	19.208	87.538
Pulticub km 44	1. Oct 15	Sian Ka´an	19.073	87.559
Banco Material Camino Pulticub	1. Oct 15	Sian Ka´an	19.076	87.578
Aguada límite de la reserva	3. Oct 15	Sian Ka´an	19.710	87.828
Vigía Chico	4. Oct 15	Sian Ka´an	19.784	87.608
Savannah km 10	5. Oct 15	Sian Ka´an	19.875	87.717
Playón	6. Oct 15	Sian Ka´an	19.824	87.503
Savannah camino Playón	6. Oct 15	Sian Ka´an	19.832	87.542
Well Bajo Savannah 2	6. Oct 15	Sian Ka´an	19.795	87.706
Savannah 2	7. Oct 15	Sian Ka´an	19.799	87.701
